# Automated classifiers for early detection and diagnosis of retinopathy in diabetic eyes

**DOI:** 10.1186/1471-2105-15-106

**Published:** 2014-04-12

**Authors:** Gábor Márk Somfai, Erika Tátrai, Lenke Laurik, Boglárka Varga, Veronika Ölvedy, Hong Jiang, Jianhua Wang, William E Smiddy, Anikó Somogyi, Delia Cabrera DeBuc

**Affiliations:** 1 Department of Ophthalmology, Faculty of Medicine, Semmelweis University, Budapest, Hungary; 2 Miller School of Medicine, Bascom Palmer Eye Institute, University of Miami, 1638 NW Tenth Avenue, Miami, FL 33136, USA; 3 2nd Department of Internal Medicine, Faculty of Medicine, Semmelweis University, Budapest, Hungary

## Abstract

**Background:**

Artificial neural networks (ANNs) have been used to classify eye diseases, such as diabetic retinopathy (DR) and glaucoma. DR is the leading cause of blindness in working-age adults in the developed world. The implementation of DR diagnostic routines could be feasibly improved by the integration of structural and optical property test measurements of the retinal structure that provide important and complementary information for reaching a diagnosis. In this study, we evaluate the capability of several structural and optical features (thickness, total reflectance and fractal dimension) of various intraretinal layers extracted from optical coherence tomography images to train a Bayesian ANN to discriminate between healthy and diabetic eyes with and with no mild retinopathy.

**Results:**

When exploring the probability as to whether the subject’s eye was healthy (diagnostic condition, Test 1), we found that the structural and optical property features of the outer plexiform layer (OPL) and the complex formed by the ganglion cell and inner plexiform layers (GCL + IPL) provided the highest probability (positive predictive value (PPV) of 91% and 89%, respectively) for the proportion of patients with positive test results (healthy condition) who were correctly diagnosed (Test 1). The true negative, TP and PPV values remained stable despite the different sizes of training data sets (Test 2). The sensitivity, specificity and PPV were greater or close to 0.70 for the retinal nerve fiber layer’s features, photoreceptor outer segments and retinal pigment epithelium when 23 diabetic eyes with mild retinopathy were mixed with 38 diabetic eyes with no retinopathy (Test 3).

**Conclusions:**

A Bayesian ANN trained on structural and optical features from optical coherence tomography data can successfully discriminate between healthy and diabetic eyes with and with no retinopathy. The fractal dimension of the OPL and the GCL + IPL complex predicted by the Bayesian radial basis function network provides better diagnostic utility to classify diabetic eyes with mild retinopathy. Moreover, the thickness and fractal dimension parameters of the retinal nerve fiber layer, photoreceptor outer segments and retinal pigment epithelium show promise for the diagnostic classification between diabetic eyes with and with no mild retinopathy.

## Background

Artificial neural networks (ANNs) have been widely used in both modern industries and scientific research to perform diverse and sophisticated tasks, such as data processing, pattern recognition, system controls and medical diagnosis [1-4]. In the field of medical diagnosis, ANNs have been widely applied in different areas of medical diagnosis, including cardiology, oncology, radiology and ophthalmology [5-8]. Because of the prediction capability of ANNs, they can be used to diagnose diseased subjects in clinical practice. The basic idea is to compare the measured target features with the predicted target features using a trained ANN that was specifically designed for a particular type of patient group. The results from comparisons using one criterion could determine whether the questionable subjects have a disease or not. With multiple criteria, ANNs could classify the questionable subjects according to differences in disease type or disease stage. In general, criteria are defined as statistically determined values or ranges that represent typical disease characteristics. The prediction and classification performed by ANNs could save doctors and patients time by determining the diagnosis of the questionable subjects in advance of treatments. The use of ANNs could improve overall positive predictive performance and reduce diagnostic time and medical costs as well as to increase the quality and accessibility of preventive care for individuals with diabetes. However, it should be noted that the costs of medical devices used in the implementation of ANNs should be taken into account as a potential limiting factor to their accessibility.

In ophthalmology, the detection of functional vision abnormalities plays a fundamental role in the diagnosis of eye diseases. Such a task depends not only on the use of a variety of precise optical instruments but also on technicians who are well trained in accurate ophthalmic techniques. The use of multiple instruments and technicians could decrease measurement precision, whereas the implementation of ANNs could improve it, in addition to reducing waiting times and medical costs. Currently, most ANN mapping of the eye structure and function involves training with measurements of retinal structure and visual function. For example, Zhu et al. developed an ANN using a Bayesian radial basis function to map the structure-function relationship between the retinal nerve fiber layer and visual function in glaucoma. The results demonstrated that ANNs using a Bayesian radial basis function could effectively improve the agreement between predicted visual function and measured visual function compared with results obtained using linear regression [9]. Furthermore, Zhu et al. quantitatively evaluated the discordance between the visual function predicted by a trained ANN and the measured visual function in glaucoma. Specifically, 39% of the predicted visual function showed significant discordance with the measured visual function [10].

Aside from the prediction of visual function, these ANNs have also been used to classify eye diseases, such as diabetic retinopathy. Diabetic retinopathy (DR) is a severe and widely spread eye disease increasing in incidence as the worldwide number of patients with diabetes grows [11]. Retinopathy is not common during the first 5 years’ duration of type 1 diabetes and at least some form of DR is present after 20 years of the onset of type 2 diabetes [12]. Thus, an objective test for the early diagnosis and evaluation of treatment in DR is certainly needed in order to identify the individuals at high risk for vision-threatening problems. The role of Optical Coherence Tomography (OCT) in the assessment and management of the diabetic retina has become significant in understanding the vitreoretinal relationships and the internal architecture of the retinal structure [13].

Previous work of ANN applications in DR has demonstrated that the input feature is no longer restricted to the thickness of the retina; it can be expanded to different types of features such as the diameter of blood vessels, the radius of the corneal surface curvature and the cross-sectional area of blood vessels [14-16]. For example, Yun et al. classified the different stages of diabetic retinopathy (i.e., moderate, severe and proliferative DR) and differentiated them from the healthy retina using a three-layer backpropagation (BPA) ANN. In their method, the perimeter and area of the veins, hemorrhages and microaneurysms were extracted from retinal fundus images and used as input to the classifier. The ANN was trained with 74 subjects (20 healthy, 27 moderate, 13 severe and 27 proliferative) and was tested with 37 subjects (9 healthy, 11 moderate, 5 severe and 12 proliferative). Their system achieved a sensitivity of 90% and a specificity of 100% for the 37 test subjects [14]. Sinthanayothin et al. proposed an automated screening system to detect blood vessels in fundus images with a three-layer ANN that had 6 input neurons, 20 hidden neurons and 2 output neurons. They achieved a sensitivity of 80.21% and a specificity of 70.66% for 484 healthy retina images and 283 diabetic retinopathy images [15]. Gardner et al. developed an ANN to differentiate diabetic retinopathy patients from healthy subjects by extracting the blood vessels, exudates and hemorrhages from images captured by a fundus camera. They achieved a sensitivity of 88.4% and a specificity of 83.5% for the detection of diabetic retinopathy when 147 diabetic and 32 healthy images were used to train the backpropagation and 200 diabetic and 101 healthy images were used for testing [16].

Most current research has used blood vessels and related features extracted from fundus images to train different types of ANNs to identify diseased eyes [17-19].

Taking into account the underlying relationship between structural and optical measurements of the retinal tissue, it is possible that test measurements from OCT images based on the integration of structural and optical properties could provide more significant information and thus superior diagnostic performance for classification methods when used as input data. To the best of our knowledge, only a few studies have used the thickness measurements extracted from OCT images to train ANNs. For example, the retinal nerve fiber layer thickness was extracted from OCT images to train a relevance vector machine to predict visual function in glaucoma [20]. In addition, the structural and optical features of various intraretinal layers extracted from OCT images have been used as discriminators to differentiate diabetic eyes with and with no mild retinopathy from healthy eyes [21]. In this study, we evaluate the capability of several structural and optical features of various intraretinal layers extracted from OCT data to train an ANN to discriminate between healthy eyes and diabetic eyes with and with no mild retinopathy.

## Results

A total of 930 OCT images obtained from 155 eligible eyes of 99 participants were analyzed. The demographic and clinical characteristics of the study population are summarized in Table 1.

**Table 1:** Characteristics of the study population

**Characteristic**	**Controls**	**DM**	**MDR**
Number of Participants	41	29	29
Number of Eyes	74	38	43
Age (years, mean ± SD)	34 ± 12	35 ± 10	43 ± 17
Female, N (% total eyes)	52 (70%)	20 (53%)	21 (49%)
Race (% Caucasian)	100	100	91
Hemoglobin A1c level (%)	-	7.20 ± 0.90	8.51 ± 1.76
DM duration (years, mean ± SD)	-	13 ± 5	22 ± 10
IOP (mmHg, mean ± SD)	-	15.74 ± 1.77	15.09 ± 1.56
BCVA	1.00 ± 0.00	1.00 ± 0.00	0.97 ± 0.06
Total macular thickness	324.36 ± 10.27	316.72 ± 21.56	297.40 ± 21.79

*Abbreviations*: *SD* standard deviation, *NA* not applicable, *DM* diabetic eyes without retinopathy, *MDR* diabetic eye with mild diabetic retinopathy.

The performance of the proposed methodology is measured using sensitivity, specificity, and positive predictive values as figures of merit. Results for true positive (TP), false negative (FN), true negative (TN), false positive (FP), positive predictive value (PPV), sensitivity and specificity in Test 1 were calculated to evaluate the classifications (see Tables 2 and 3). In this classification test, we explored the probability as to whether the subject’s eye was healthy (diagnostic condition). Table 2 shows the sensitivity, specificity, predictive values and positive predictive values obtained when training the Bayesian radial basis function network using the thickness (TH) and fractal dimension (FD) as the input and target features of the retinal layers, respectively. Our results indicated that the TP test for the healthy eyes was in the [48–51] range when 54 healthy eyes were mixed with 43 diabetic eyes with mild retinopathy (MDR) in this test. Particularly, TP achieved high values (49, 50 and 51, respectively) for OCT parameters of the GCL + IPL complex, OS and RPE. As indicated by the positive predictive values, a high probability was achieved for the GCL + IPL complex and OPL parameters (91% and 89%, respectively) indicating that the subject really has a healthy eye. The TN test was in the [9–36] range and high TN values (35 and 36, respectively) were achieved for the GCL + IPL complex and OPL features used in this particular tests. Moreover, high values for sensitivity, specificity and PPV (≥0.80) were only obtained for the GCL + IPL complex and OPL parameters.

**Table 2:** Test classification performance results obtained in Test 1

**TH vs. FD**	**RNFL (eye/scans)**	**GCL + IPL (eye/scans)**	**INL (eye/scans)**	**OPL (eye/scans)**	**ONL + IS (eye/scans)**	**OS (eye/scans)**	**RPE (eye/scans)**
**TP**	48/288	49/294	48/288	48/288	48/288	50/300	51/306
**FN**	6/36	5/30	6/36	6/36	6/36	4/24	3/18
**TN**	10/60	35/210	23/138	36/216	10/60	9/54	11/66
**FP**	33/198	8/48	20/120	7/42	33/198	34/204	32/192
**PPV**	0.59	0.86 *	0.71	0.87 *	0.59	0.60	0.61
**Sensitivity**	0.89	0.91*	0.89	0.89*	0.89	0.93	0.94
**Specificity**	0.23	0.81*	0.53	0.84*	0.23	0.21	0.26

*denotes the intraretinal layer for which the sensitivity, specificity and PPV are greater than 80%.

Sensitivity, specificity, predictive values (TP, FN, TN, FP) and positive predictive values (PPV) obtained when training the Bayesian radial basis function network using the thickness (TH) and fractal dimension (FD) as the input and target features of the given retinal layers, respectively.

**Table 3:** Test classification performance results obtained in Test 1 after using the total reflectance as an input feature

**TR vs. FD**	**RNFL (eye/scans)**	**GCL + IPL (eye/scans)**	**INL (eye/scans)**	**OPL (eye/scans)**	**ONL + IS (eye/scans)**	**OS (eye/scans)**	**RPE (eye/scans)**
**TP**	48/288	49/294	48/288	48/288	48/288	50/300	51/306
**FN**	6/36	5/30	6/36	6/36	6/36	4/24	3/18
**TN**	10/60	35/210	23/138	37/222	9/54	9/54	11/66
**FP**	33/198	8/48	20/120	6/36	34/204	34/204	32/192
**PPV**	0.59	0.86	0.71	0.89	0.59	0.60	0.61
**Sensitivity**	0.89	0.91*	0.89	0.89*	0.89	0.93	0.94
**Specificity**	0.23	0.81*	0.53	0.86*	0.21	0.21	0.26

*denotes the intraretinal layer for which the sensitivity, specificity and PPV are greater than 80%.

Sensitivity, specificity, predictive values (TP, FN, TN, FP) and positive predictive values (PPV) obtained when training the Bayesian radial basis function network using the total reflectance (TR) and fractal dimension (FD) as the input and target features, respectively.

Table 3 shows the sensitivity, specificity, predictive values and positive predictive values obtained when training the Bayesian radial basis function network using the total reflectance and fractal dimension as the input and target features, respectively. Our results indicated that the TP and TN tests for healthy eyes were in the [48–51] and [9–36] ranges; respectively. As indicated by the positive predictive values, a high probability was achieved for the features of the GCL + IPL complex and OPL (91% and 89%, respectively) indicating that the subject really has a healthy eye. Specifically, high TN values (35 and 36, respectively) were achieved for the parameters of the GCL + IPL complex and OPL. Moreover, high values for sensitivity, specificity and PPV (≥0.80) were only obtained for the features of the GCL + IPL complex and OPL. Therefore, there is high probability (≥80%) the subject will have a healthy GCL + IPL complex and OPL structure.

Tables 4 and 5 show results obtained after using different sizes of training data sets (20, 30 and 40 healthy eyes, respectively) in Test 2. When training the Bayesian radial basis function network using the thickness (total reflectance) and fractal dimension as the input and target features, our results demonstrated that the FN and FP values remaining at a given sensitivity of ≥ 80% for the GCL + IPL complex’s parameters were stable despite the amount of healthy eyes used in the training task, whereas the values of FN remaining for the OPL were slightly reduced with the increased number of healthy eyes used to train the ANN. Additionally, the TN value for the parameters of the GCL + IPL complex was stable. Our results showed relatively high PPV, as well as high sensitivity and specificity (≥0.80) in both the GCL + IPL complex and OPL’s parameters. Our results showed that PPV had a slight decreasing trend for both the GCL + IPL complex and OPL’s parameters when the number of healthy subjects increased from 20 to 40 in the training task, which was due to a decrease in test subjects (healthy eyes).

**Table 4:** Model testing results obtained after changing the size of the training data set

**Size of the training data set**	**20 healthy eyes**		**30 healthy eyes**		**40 healthy eyes**	
**TH vs. FD**	**GCL + IPL (eye/scans)**	**OPL (eye/scans)**	**GCL + IPL (eye/scans)**	**OPL (eye/scans)**	**GCL + IPL (eye/scans)**	**OPL (eye/scans)**
**TP**	49/294	48/288	39/234	39/234	29/174	29/174
**FN**	5/30	6/36	5/30	5/30	5/30	5/30
**TN**	35/210	36/216	35/210	36/216	35/210	36/216
**FP**	8/48	7/42	8/48	7/42	8/48	7/42
**PPV**	0.86	0.87	0.83	0.85	0.78	0.81
**Sensitivity**	0.91	0.89	0.89	0.89	0.85	0.85
**Specificity**	0.81	0.84	0.81	0.84	0.81	0.84

Results of sensitivity, specificity, accuracy, predictive values and positive predictive values obtained for the GCL + IPL complex and OPL when training the Bayesian radial base function network with 20, 30 and 40 healthy eyes with the thickness (TH) and fractal dimension (FD) as the input and target features, respectively.

**Table 5:** Model testing results obtained after changing the size of the training data set and using the TR as an input feature

**Size of the training data set**	**20 healthy eyes**		**30 healthy eyes**		**40 healthy eyes**	
**TR vs. FD**	**GCL + IPL (eye/scans)**	**OPL (eye/scans)**	**GCL + IPL (eye/scans)**	**OPL (eye/scans)**	**GCL + IPL (eye/scans)**	**OPL (eye/scans)**
**TP**	49/294	48/288	39/234	39/234	29/174	29/174
**FN**	5/30	6/36	5/30	5/30	5/30	5/30
**TN**	35/210	37/222	35/210	36/216	35/210	37/222
**FP**	8/48	6/36	8/48	7/42	8/48	6/36
**PPV**	0.86	0.89	0.83	0.85	0.78	0.83
**Sensitivity**	0.91	0.89	0.89	0.89	0.85	0.85
**Specificity**	0.81	0.86	0.81	0.84	0.81	0.86

Results of sensitivity, specificity, accuracy, predictive values and positive predictive values obtained for the GCL + IPL complex and OPL when training the Bayesian radial basis function network with 20, 30 and 40 healthy eyes with the total reflectance (TR) and fractal dimension (FD) as the input and target features, respectively.

Results obtained in Test 3 after training the Bayesian radial basis function network with the thickness measurement and fractal dimension as the input and target features are shown in Table 6. In this classification test, we explored the probability as to whether a diabetic eye had MDR (diagnostic condition). Our results indicated high TP values for features of the RNFL, GCL + IPL complex, OS and RPE. Additionally, the sensitivity, specificity and positive predicted values were greater or close to 0.70 in the RNFL, OS and RPE. Interestingly, the GCL + IPL complex’s features didn’t show a PPV greater than 80%.

**Table 6:** Test classification performance results obtained in Test 3

**TH vs. FD**	**RNFL (eye/scans)**	**GCL + IPL. (eye/scans)**	**INL (eye/scans)**	**OPL (eye/scans)**	**ONL + IS (eye/scans)**	**OS (eye/scans)**	**RPE (eye/scans)**
**TP**	18/108	18/108	15/90	4/24	10/60	18/108	20/120
**FN**	5/30	5/30	8/48	19/114	13/78	5/30	3/18
**TN**	30/180	26/156	32/192	28/168	26/162	31/186	33/198
**FP**	8/48	12/72	6/36	10/60	12/72	7/42	5/30
**PPV**	0.69	0.60	0.71	0.29	0.45	0.72	0.80*
**Sensitivity**	0.78	0.78	0.65	0.17	0.43	0.78	0.87*
**Specificity**	0.79	0.68	0.84	0.74	0.68	0.82	0.87*

*denotes the intraretinal layer for which the sensitivity, specificity and PPV are greater than 80%.

Sensitivity, specificity, predictive values (TP, FN, TN, FP) and positive predictive values (PPV) obtained when training the Bayesian radial basis function network using the thickness (TH) and fractal dimension (FD) as the input and target features, respectively.

In general, the overall results indicate that the classifier is effective to about 90 per cent (PPV values in Tables 3 and 4) in making the correct prediction of the unknown class (healthy eyes) when differentiating healthy from MDR eyes by using the features of the GCL + IPL complex and OPL in the diagnostic test (Test 1). However, the classifier was not effective (~44.5%) in making the correct prediction of the unknown class (MDR eyes) when discriminating between DM and MDR eyes using the same intraretinal layer’s features (i.e. GCL + IPL complex and OPL in Test 3). Interestingly, the classifier was more effective (PPV ~ 74%) in making the correct prediction of the unknown class (MDR eyes) when differentiating DM and MDR eyes by using the features of the RNFL, OS and RPE in the diagnostic test (Test 3). Table 7 shows the percentage of correct classifications for the GCL + IPL complex and OPL features in tests 1 and 3.

**Table 7:** Percentage of correct classifications as a function of eyes used in training and testing in tests 1 and 3

**Intraretinal Layer**	**Number of eyes used for training**		**Number of eyes used for testing**	**Percentage of correct classifications (%)**
GC + IPL	Test 1	20 Healthy	97	91
	Test 3	20 MDR	61	42
OPL	Test 1	20 Healthy	97	89
	Test 3	20 MDR	61	47

## Discussion

In this study, we presented and evaluated a nonlinear prediction method for early retinopathy detection on OCT retinal images. The proposed system consisted of three phases: preprocessing and image segmentation, candidate MDR feature detection, and feature set formulation and classification. We have used sensitivity, specificity, predictive values (TP, TN, FP, FN) and PPV parameters to measure the classification performance of the ANN ensemble and the diagnostic ability of the integrated OCT parameters. Quantitative tools for measuring thickness information of the retinal tissue using OCT devices are in common clinical use, but to our knowledge there have been no algorithms available to analyze the optical properties of the retinal tissue and further combine them with structural information to assess the integrity and better predict the lack of integrity of the retinal layers in diabetic eyes. The use of the predictability of retinal layer integrity’s loss from structural and optical features by the Bayesian radial basis function network played a key role in the neural loss assessment in diabetic eyes. In our proposed method, the stable trend of the FN values (of healthy testing eyes in Test 2) validated the reliability of the methodology. Our results demonstrate that the GCL + IPL complex and OPL parameters could be predicted and used to discriminate between MDR and healthy eyes by using either the TH/FD or TR/FD pairs as the input/target features in the Bayesian radial basis function network. The high sensitivity and specificity values obtained when using structural and optical parameters of the GCL + IPL complex and OPL suggest that the Bayesian radial basis function network can be used to discriminate between MDR and healthy eyes with the selected input and target features extracted from OCT images. In particular, the fractal dimension, which represents the roughness of the intraretinal layer structure, could certainly be used to differentiate MDR from healthy eyes. Our results suggest that the GCL + IPL complex and OPL are more susceptible to early damage in MDR eyes. The low RNFL specificity and PPV values indicated that RNFL parameters were not good input/output targets for use in ANNs to differentiate between MDR and healthy eyes. Interestingly, the features of the RNFL, OS and RPE better predicted the lack of integrity of the retinal structure when discriminating between MDR and DM eyes. This particular result is in agreement with previous studies reporting changes in the outer retinal segment when comparing the macular thickness in diabetic subjects with mild retinopathy and healthy eyes [22,23]. The above finding may prove to be useful for the better detection of mild diabetic retinopathy by using optical coherence tomography imaging.

There were some limitations in this study. First, comparisons across studies were not possible, because no studies have been conducted to investigate thickness and optical properties of the retinal tissue together, using ANNs. Second, larger sample sizes would provide more accurate and robust estimations of the classification test performance. However, our results can be used as the basis for further improving the diagnostic accuracy of early DR detection in the near future. Third, the specific automated classification method that we chose is likely not to be the only one that could be applied. Comparisons among other automated classification methods should be made to obtain the best models for improving the discriminant power of OCT integrated data for parameter tests in decision support systems.

As already established, a Bayesian radial basis function network can accommodate uncertainty in the dimension of the model by adjusting the sizes to the complexity of the data [24]. In this study, the TN, TP and the PPV values remained stable despite the different sizes of training data sets. However, training the Bayesian radial basis function network may require more test subjects, which would improve the precision of the differentiation between healthy eyes and diabetic eyes with and without mild retinopathy. Future studies should also evaluate the methodology with data based on the new generation of OCT devices that provide higher spatial resolution for analyzing the retinal structure.

## Conclusions

In this study, we have employed for the first time a method that uses a Bayesian ANN with four pairs of input and target features extracted from OCT data to discriminate among MDR, healthy and DM eyes. The input features used were the intraretinal layer thickness measurement and total reflectance extracted from OCT images. The fractal dimension of the GCL + IPL complex and OPL predicted by the Bayesian radial basis function network positively discriminated between MDR and healthy eyes. Moreover, the thickness and fractal dimension parameters of the RNFL, OS and RPE show promise for diagnostic classification between MDR and DM eyes. The results demonstrated that the proposed Bayesian radial basis function network’s classification can be used in a computer-aided diagnostic system for discriminating between healthy eyes and diabetic eyes with early retinopathy as it identified and detected retinal features with high probability for the proportion of patients with positive test results who were correctly diagnosed. Our study showed that the combination of structural and optical information from OCT data has the potential to improve parameter tests that better reflect the diabetic retinal changes that occur during the progression of the disease, providing more relevant information to DR diagnostic routines. Such improvements could facilitate the practical implementation of ANNs as decision support systems in DR diagnostics.

## Methods

A total of 120 participants (190 eyes) were recruited between October 2007 and December 2010 at the Department of Ophthalmology, Semmelweis University, Budapest, Hungary under a Juvenile Diabetes Research Foundation study. The research adhered to the tenets set forth in the declaration of Helsinki. Instutional Review Board approval was obtained both at Semmelweis University and the Miller School of Medicine, University of Miami. In this prospective study, enrollment was offered to all Type 1 diabetic patients referred to the comprehensive ophthalmology clinic that had diabetic retinopathy up to ETDRS level 35 without macular edema, as well as diabetic patients with no retinopathy [25,26]. Moreover, we did not include patients with proliferative disease, clinically significant macular edema (CSME) and with anatomic abnormalities that could distort macular architecture, such as glaucoma, vitreoretinal traction and epiretinal membranes. We enrolled only patients over the age of 18 and written informed consent was obtained from each subject. OCT examination was performed in healthy and diabetic eyes with and with no retinopathy.

Once the subject was enrolled in the study, only one visit was required to perform a comprehensive eye examination including intraocular pressure (using Goldmann tonometer) and slit-lamp examination. Fundus images were obtained and classified by an experienced grader according to the criteria of the ETDRS protocol [23]. The grader classified images without being aware of the OCT findings and clinical data. In addition, a hemoglobin A1c level test was required at this visit for diabetic patients with no past glycemic control. No additional tests were required after this primary visit and during the time the study was completed. Inclusion criteria for healthy controls included best-corrected visual acuity of 20/25 or better, no history of any current ocular or systematic disease, and a normal appearing macula on contact lens biomicroscopy. Patients with any medical condition that might affect visual function other than type 1 diabetes, or treatments with medications that might affect retinal thickness were excluded from the study. Moreover, patients who have recently undergone cataract surgery, or with any history of intraocular surgery, and patients with currently unstable blood sugars or who have recently been placed on insulin pump therapy were also excluded from the study.

Thirty five eyes of 21 participants were excluded because of low quality OCT scans (1) and other diseases listed under the exclusion criteria (amblyopic (3), chorioretinitis (2), moderate DR (6), no DR (2), epiretinal membrane (1), panretinal photocoagulation (5), pars plana vitrectomy & panretinal photocoagulation (1), pigment epithelial detachment & central serous chorioretinopathy (1), type 2 DM (8), optic nerve disease (3), and severe DR (2)). The remaining 155 eligible eyes from 99 participants were analyzed, which included a total of 74 healthy eyes (34 ± 12 yrs, 52 female, 22 male), 38 eyes with type 1 diabetes mellitus (DM) with no retinopathy (35 ± 10 yrs, 20 female, 18 male) and 43 eyes with mild diabetic retinopathy (MDR, 43 ± 17 yrs, 21 female, 22 male) on biomicroscopy were included in the study (see Table 1).

The OCT system (Stratus OCT, Carl Zeiss Meditec, Dublin, California) used in this study employs a broadband light source, delivering an output power of 1 mW at the central wavelength of 820 nm with a bandwidth of 25 nm. The light source yields 12 μm axial resolution in free space that determines the imaging axial resolution of the system. A cross-sectional image is achieved by the combination of axial reflectance while the sample is scanned laterally. All Stratus OCT study cases were obtained using the macular thickness map protocol. This protocol consists of six radial scan lines centered on the fovea, each having a 6 mm transverse length. In order to obtain the best image quality, focusing and optimization settings were controlled and scans were accepted only if the signal strength was above 6 (preferably 9–10) [27]. Scans with foveal decentration (i.e. with center point thickness SD > 10%) were repeated.

**Figure 1:**
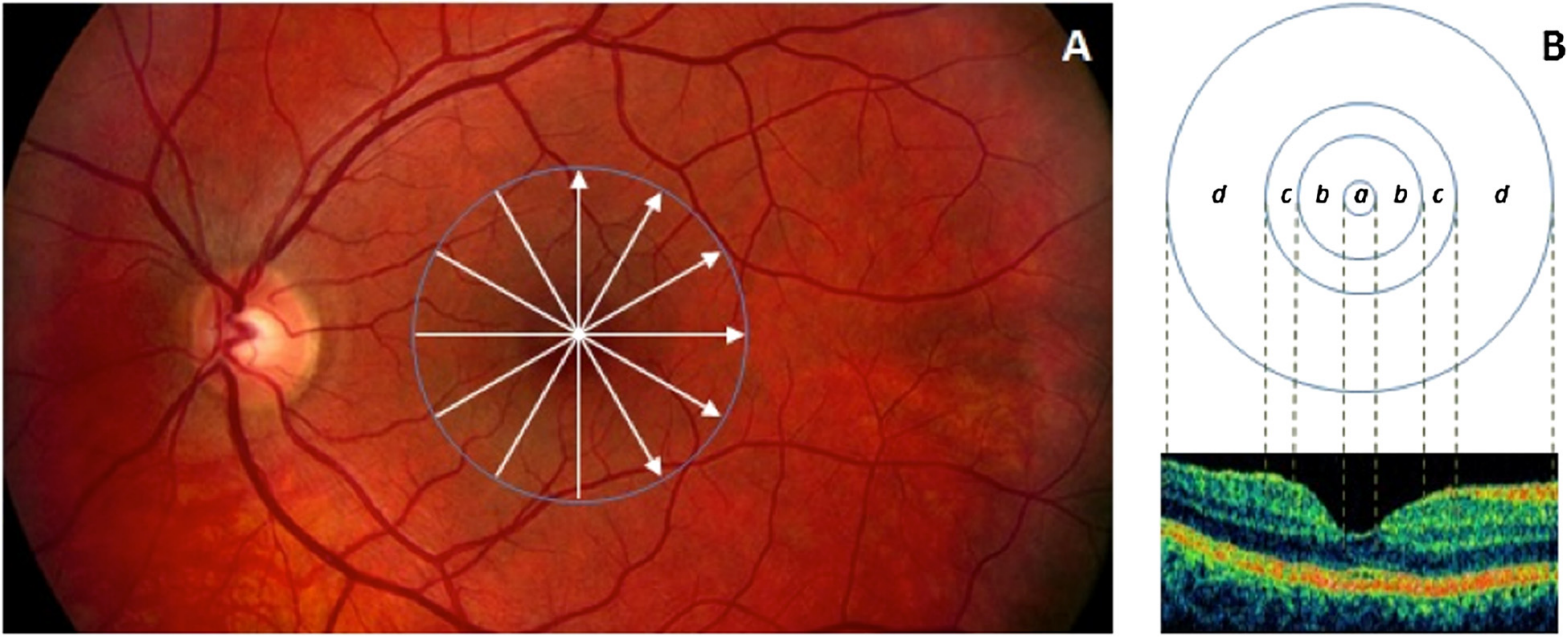
**Custom-built method showing macular sectors. A)** Fundus image of a healthy eye showing the Stratus OCT’s radial lines protocol. **B)** Regions shown are: foveola (a) with a diameter of 0.35 mm, foveal region (b) with a diameter of 1.85 mm, parafoveal region (c) with a diameter of 2.85 mm and perifoveal (d) region with a diameter of 5.85 mm.

Macular radial line scans of the retina for each case were exported to disc with the export feature available in the Stratus OCT device and analyzed using a custom-built software (OCTRIMA) [28]. A total of 6 cellular layers of the retina were segmented on OCT images based on their optical densities: the retinal nerve fiber layer (RNFL), the ganglion cell and inner plexiform layer complex (GCL + IPL), the inner nuclear layer (INL), the outer plexiform layer (OPL), the outer nuclear layer and inner photoreceptor segment (ONL + IS), outer photoreceptor segment (OS) and retinal pigment epithelium (RPE) (see Figure 1) [28]. As in some Fourier-domain OCT (FD-OCT) systems, OCTRIMA facilitates the total retinal thickness calculations between the ILM and the inner boundary of the second hyperreflective band, which has been attributed to the outer segment/retinal pigment epithelium (OS/RPE) junction in agreement with histological and previous OCT studies [29-32]. Structural and optical measurements, in addition to thickness measurements, were extracted using features measured locally for each intraretinal layer. The image processing and diagnostic parameter calculations were programmed in Matlab 7.0 (The Mathworks, Natick, Massachusetts).

The macular region was divided into separate regions (see Figure 1). The central disc is the foveola area with a diameter of 0.35 mm. The remaining rings are the fovea, parafoveal and perifoveal areas with a diameter of 1.85, 2.85 and 5.85 mm, respectively. Because an area with a diameter of 1 mm is too large for the thickness of the foveola region, which is only approximately 0.35 mm in diameter, the custom-built map allows collection of more precise information near the foveola region compared to the ETDRS thickness map. In addition, no interpolation is used in this method.

Structural and optical properties, in addition to thickness measurements, were extracted from OCT-based images and were used for the classification of healthy eyes and diabetic eyes with and with no retinopathy [21,33]. The structural and optical parameters that were best able to discriminate between diabetic eyes and healthy eyes, as revealed by statistical and receiver operating characteristic analyses from previous work [21,33], were evaluated and validated by artificial neural networks with a Bayesian radial basis function [24].

Our ANN classifier consisted of an ensemble of two input neurons with a Bayesian radial basis function and one output neuron. Therefore for each candidate intraretinal layer we have two features (input parameters) that are fed into the ANN to predict one output feature in each classification test. The ANNs were implemented in Matlab 7.0 (The Mathworks, Natick, Massachusetts) using Markov chain Monte Carlo (MCMC) algorithms. In order to cancel out interdata variations, a correlation matrix based on standardized values of all parameters was used in our study. Therefore, each dataset’s feature was normalized to have zero mean and unit variance by dividing the mean corrected data by the respective SD before further processing. The relative error *ϵ*_*γ*_ between the predicted and measured values was used to evaluate the predicted values (see Eq.1).

(1) $$\epsilon_{\gamma }=\left\{V_{\gamma }-V\right\}/V$$

where *V* denotes the measured values of the output parameters extracted from the unknown subjects and V_p_ denotes the predicted values of the output parameters. The distribution of the relative errors E_p_ was assumed to be the Gaussian function (see Eq.2),

(2) $$f\left(x\right)=\frac{1}{\sqrt{2\pi \sigma^{2}}}c-\frac{{\left(x-\mu \right)}^{2}}{2\sigma^{2}}$$

where μ μ is the average value of *ϵ*_*γ*_; σ is the deviation of *ϵ*_*γ*_. Then, a proper positive parameter *c*_*y*_ was used to define the range [*μ* - *c*_*ρ*_*σ*, *μ* + *c*_*ρ*_*σ*]. By integrating the Gaussian function within this range, the Gaussian error function was calculated as:

(3) $$\begin{array}{ll} \;S\left(c_{\rho }\right) & =\int_{\mu -c_{\rho }\sigma }^{\mu +c_{\rho }\sigma }f\left(x\right)\mathrm{dx}=\int_{\mu -c_{\rho }\sigma }^{u+c_{\rho }\sigma }\frac{1}{\sqrt{2\pi \sigma^{2}}}c-\frac{{\left(x-\mu \right)}^{2}}{2c^{2}}\mathrm{dx}\\ & =\mathrm{crf}\left(c_{\gamma }/\sqrt{2}\right) \end{array}$$

The value of the Gaussian error function (*c*_*y*_) reflects the possibility ratio of the set of relative errors *ϵ*_*γ*_ in the range [*μ* - *c*_*ρ*_*σ*, *μ* + *c*_*ρ*_*σ*]. A series of typical values of [*c*_*ρ*_*S*(*cγ*)] is listed in Table 8. In this study, the parameter *c*_*y*_ was initialized as 1.65, which yielded 90% accuracy for the classification. Once the parameter *c*_*y*_ was obtained from the training set used for training the Bayesian radial basis function network, the discrimination task was performed on all subjects by comparing the measured values and the predicted values using the Bayesian radial basis function network.

**Table 8:** **Typical values of c**_**p **_**and Gaussian error function**

***c***_***v***_	**s(c**_**p**_**)**
1.28	80%
1.44	85%
1.65	90%
1.96	95%
2.58	99%

Different training and classification tasks for discriminating between diabetic and healthy eyes were performed. Particularly, structural and optical parameters of intraretinal layers were chosen as the input and output features for the Bayesian radial basis function networks that would discriminate among MDR, healthy and DM eyes. As indicated in previous work [21], thickness measurement (TH), fractal dimension (FD) and total reflectance (TR) showed better discrimination power than other parameters among MDR, healthy and DM eyes. Therefore, these three optimum parameters were used as the input and output values required in the training task of Bayesian radial basis function networks. Then, trained Bayesian radial basis function networks were used to classify the mixed test subjects (excluding the training subjects). To explore the probabilistic relationships between the diabetic retinal disease and target features (i.e., symptoms), we first performed the training task using a subset of the data and different pairs of input and output target features. Then, classification tasks were performed to obtain the optimum distribution over the set of allowed models. Additionally, a classification test’s performance as a function of training set size was used to assess adequacy of the training data set in the development of the ANN scheme. Therefore, different sizes of the training set were explored and the corresponding results were compared. Specifically, we first explored the probabilistic relationships between the diabetic retinal disease and target features. Particularly, a total of 20 healthy eyes were randomly selected from the healthy group (out of 74 healthy eyes) to train the Bayesian radial basis function network (Test 1). Different pairs of input and target features extracted from all intraretinal layers were used to train the Bayesian radial basis function network and to classify a total of 43 MDR eyes using the remaining 54 healthy eyes (not used in training) from the healthy group. In this test, we evaluated the feasibility of the method and determine the best intraretinal layer parameters that could be predicted and used to discriminate between MDR and healthy eyes. Second, we performed model testing of the previous experiment by exploring different sizes of the training data subset (Test 2). In this second test, different sizes of the training data subset (20, 30 and 40 healthy eyes) were chosen to train the Bayesian radial basis function network and corresponding results were compared. Then, we tried to discriminate between DM and MDR eyes (Test 3). As in the previous test, 20 MDR eyes were randomly selected from the total 43 MDR eyes to train the Bayesian radial basis function network with the TH/FD and TR/FD as the input and target features, respectively. Then, the trained Bayesian radial basis function network was used to classify the remaining 23 MDR eyes and 38 DM eyes.

## Abbreviations

OCT: Optical coherence tomography; MDR: Mild diabetic retinopathy; OCTRIMA: OCT retinal image analysis; RNFL: Retinal nerve fiber layer; GCL + IPL: Ganglion cell and inner plexiform layer complex; INL: Inner nuclear layer; OPL: Outer plexiform layer; ONL: Outer nuclear layer; ONL + IS: Complex formed by the outer nuclear layer and the inner photoreceptor segment; OS: Outer photoreceptor segment; RPE: Retinal pigment epithelium; FD: Fractal dimension; SD: Standard deviation.

## Competing interests

The University of Miami and Dr. Cabrera DeBuc hold a pending patent used in the study and have the potential for financial benefit from its future commercialization. All other authors of the paper report no disclosures.

## Authors’ contributions

GMS, ET, WES and AS collected clinical data. GMS, ET, RT, LL, BV, VÖ analyzed the data. DCD, JH, GMS and JW interpreted data. GMS and DCD drafted the manuscript. DCD and GMS supervised this study. DCD designed the study. All authors read and approved the final manuscript.
